# Genetic conflict between sexual signalling and juvenile survival in the three-spined stickleback

**DOI:** 10.1186/s12862-016-0613-4

**Published:** 2016-02-29

**Authors:** Sin-Yeon Kim, Alberto Velando

**Affiliations:** Departamento de Ecoloxía e Bioloxía Animal, Universidade de Vigo, Vigo, 36310 Spain

**Keywords:** Antagonistic pleiotropy, Animal model, Carotenoid, Genetic correlation, Good genes, Sexual conflict, Sexual trait, Survival

## Abstract

**Background:**

Secondary sexual traits and mating preferences may evolve in part because the offspring of attractive males inherit attractiveness and other genetically correlated traits such as fecundity and viability. A problem regarding these indirect genetic mechanisms is how sufficient genetic variation in the traits subject to sexual selection is maintained within a population. Here we explored the additive genetic correlations between carotenoid-based male ornament colouration, female fecundity and juvenile survival rate in the three-spined stickleback (*Gasterosteus aculeatus*) to test the possibility that attractiveness genes reduce important fitness components in the bearers not expressing the sexual trait.

**Results:**

Male sexual attractiveness (i.e., red nuptial colouration) as well as female fecundity and juvenile viability showed heritable variations in the three-spined stickleback. Thus, females can gain indirect benefits by mating with an attractive male. There was a strong positive genetic correlation between female fecundity and juvenile viability. However, red sexual signal of male sticklebacks was negatively genetically correlated with juvenile survival, suggesting genetic conflict between attractiveness and viability. There was no significant correlation between attractiveness of brothers and fecundity of sisters, suggesting no intra-locus sexual conflict.

**Conclusions:**

The negative effects of mating with a colourful male on offspring viability may contribute to maintaining the heritable variation under strong directional sexual selection. The strength of indirect sexual selection may be weaker than previously thought due to the hidden genetic conflicts.

**Electronic supplementary material:**

The online version of this article (doi:10.1186/s12862-016-0613-4) contains supplementary material, which is available to authorized users.

## Background

Understanding the evolution of male sexual traits and female mating preferences has been a long-standing task in evolutionary biology since C Darwin [1]. It is generally thought that sexually selected traits evolve because non-random mating brings direct or indirect benefits to females [2, 3]. Female preference can evolve under natural selection for either direct phenotypic benefits associated with a male ornament, such as a high quality territory, nutrition, parental care or protection, [4] or other reasons, such as sensory biases [5]. On the other hand, indirect genetic benefits arise when the offspring of attractive males inherit attractiveness (“Fisherian sexy sons” [6]) and/or other genetically correlated traits such as fecundity and viability (“good genes” [7, 8]). Substantial effort has been devoted to hypothesising and testing indirect genetic mechanisms that drive the evolution of ornamental traits and associated mating preferences [9]. Nevertheless, there remain problems regarding the maintenance of sufficient genetic variation in these traits in order to sustain female choice through indirect genetic benefits (“the paradox of the lek” [10–12]).

Indeed how to explain the maintenance of genetic variation against the eroding effects of selection is a central problem in current evolutionary biology [13–15]. This problem is based on the fact that fitness is always under directional selection, and thus a single best genotype should become predominant within a population [16]. Persistent female preferences for elaborate male ornaments should erode genetic variance in these traits, eventually eliminating any indirect genetic benefit to the preferences. Numerous solutions to the maintenance of genetic variance under sexual selection have been proposed, including the capture of genetic variance by condition dependent traits and indirect genetic effects [17–19]. Antagonistic pleiotropy that constrains a sexually selected trait may be also an important mechanism. The intra-locus sexual conflict, in particular, is produced by antagonistic selection, in which favourable alleles for male fitness are detrimental for female fitness (e.g., [20, 21]). Antagonistic pleiotropy may also arise between sexually selected traits and life-history traits, both of which are most closely related to fitness, but few studies provide empirical support for this mechanism [22, 23].

A large number of sexually selected coloured ornaments in animals are based on carotenoids, which they cannot synthesise *de novo* but acquire from their diet [24]. Carotenoids also have several physiological functions including modulation of the immune system [25] and protection of soma and developing sperm against oxidative damage [26, 27]. Therefore, a carotenoid-based signal has the potential to be an honest indicator of quality but at the expense of trade-offs with these other critical functions [28, 29]. In birds, for example, the positive association between conditions experienced in early life and the expression of carotenoid-based sexual traits [30, 31] in turn suggests a trade-off between the physiological functions during development and the later expression of sexual ornaments.

In this study, we explore the genetic relationships between carotenoid-based male ornament colouration, female fecundity and juvenile survival rate in the three-spined stickleback (*Gasterosteus aculeatus*). The red ornament that stickleback males express in their cheeks and throat during the reproductive season is one of the most frequently studied sexual traits. Female sticklebacks preferentially mate with redder males [32]. The red ornament is an honest indicator of condition and parasite resistance (e.g., [33, 34]). Previous studies of the three-spined stickleback have shown that individual variation in the carotenoid signal has a strong genetic component; there is a positive genetic correlation between the red colouration and female preferences for a redder male [35–37]. Here, we test the presence of genetic conflict between ornament expression and other fitness-related traits by exploring whether the attractiveness of a male is genetically correlated with life-history traits of its family. We provide rare evidence for the negative genetic correlation between male sexual signal and juvenile viability.

## Methods

### Ethics

Wild fish were sampled under permission from the Xunta de Galicia (021/2013), and the study procedures were approved by the Animal Ethics Committee of the Universidad de Vigo (17/12 and 10/14).

### Breeding design and rearing condition

Three-spined sticklebacks were captured in the Rio Ulla (Spain) in February 2013 before sexual maturation and used for breeding. Like in other populations near the southern edge of the species’ range [38], the majority of fish in this population reproduce repeatedly throughout a single relatively long breeding season, after which they die. A total of 32 F1 families were produced by breeding16 sires and 16 dams. Each breeder mated twice with two different mates during April-May 2013 (for details, see [39]). Thus, each F1 fish had full-sibs and maternal and paternal half-sibs. The breeders were housed in individual tanks and paired with a single mate at a time. The fertilised clutches were collected from the nests within 3 h and incubated in a 100-l tank, following the standard egg husbandry protocol [40]. Each full-sib clutch was isolated in a hatching tank with a sponge filter prior to hatching; then hatchlings were reared there until age 40 days (mean ± SE number of fry per full-sib family: 57.8 ± 3.0, *n* = 32 families). Survival rate of the F1 families during the first 40 days was extremely high (mean survival rate: 0.971 ± 0.006).

At age 40 days, fry in each F1 full-sib family were allocated among two (*n* = 7 families) or four (*n* = 25 families) 8-l growth tanks (*n* = 114 tanks). Each growth tank initially housed 11 or 12 juvenile fish. The rest of the fish were housed separately and used in other studies. The growth tanks were connected to four closed water systems (30 tanks/system), in which water was continuously filtered for nitrification, aerated and temperature-controlled by the combined flow-through function. Juvenile fish were fed daily ad libitum on a progressive diet of newly hatched *Artemia* and a commercial pelleted diet (Gemma Micro, Skretting, Norway). We analysed the total carotenoid concentration in these food items. Carotenoids were repeatedly extracted using *n*-hexane, and then carotenoid concentration was determined in a spectrophotometer (Synergy HT, BioTek, Winooski, VT, USA) at 440 nm using a lutein curve as standard. Both food items contained high levels of carotenoids (wet *Artemia* larvae: 16.9 μg g^−1^; dry food pellets: 103.9 μg g^−1^). The programmed photoperiod in the tanks reflected the natural seasonal pattern in the region. This fish stock was reared also for an experiment to test reaction norms of life-history traits in response to winter temperature; a half of each F1 family replicates (i.e., growth thanks) were maintained at 14 °C and the other half experienced a gradual temperature change to 9 °C during winter [37]. This difference in winter conditions was taken into account in all statistical analyses.

At age 6 months juvenile fishes were permanently marked with colour elastomer tags (Northwest Marine Technologies, Shaw Island, WA, USA) under a low dose of benzocaine anaesthetic to track individual life-histories (*n* = 1038 individuals). Randomly selected samples from the fish stock were sacrificed with an overdose of benzocaine anaesthetic during the growth period to be used in the study of temperature manipulation (*n* = 221 in Sep-Nov 2013; *n* = 364 in Feb 2014). The juvenile survival rate was calculated in each full-sib family replicate as the proportion of individuals surviving to maturity (i.e., expression of red colouration in males and spawning in females) in the growth tank, excluding the sacrificed individuals.

### Measuring male sexual signal and female fecundity

A total of 392 F1 males sexually matured and expressed red nuptial colour in the 2014 breeding season. Among these, 209 males were randomly selected and allocated into individual tanks containing a sponge filter and nesting materials (i.e., sand and polyester thread). During 6 months (March-August) of the reproductive season, each male was shown a gravid female enclosed in a transparent glass for 5 min twice a week to prompt expression of nuptial colour, nest construction and courtship. Each male was repeatedly photographed every two weeks up to 11 times throughout the season (on average 10 times). The photographs were scheduled so that the males were always photographed 2–3 h after the stimulation with a gravid female. On each occasion the fish was placed in a small transparent water-filled plastic box, positioned on its lateral side (either left or right to reduce handling time) using a grey sponge and photographed under standardized conditions within a black box containing illumination [41]. A stickleback reduces its nuptial colour while being handled out of the water [42]. Thus, a fish was introduced to the water-filled photography box immediately after being gently netted from its home tank. The whole process away from the tank took less than 1 min. We measured the area of red nuptial colouration (hue: 1–60 and 340–359; saturation: 50–255; intensity: 0–255) from the digital images by using image analysis software (analySIS FIVE, Olympus). Relative size of the red area was calculated as a percentage of the total lateral body area for analyses. The individual seasonal maximum coloration was strongly correlated to individual mean colouration during the season (*r*^2^ = 0.728, *F*_1,207_ = 553.50, *P* < 0.001). Thus, the seasonal maximum was used for statistical analyses.

F1 females kept in their growth tanks were monitored daily to record the maturation and fecundity. The earliest started to spawn in February, and 327 females spawned repeatedly until August (*n* = 2044 clutches). Whenever a female became fully gravid, the egg clutch was stripped by applying gentle pressure to the abdomen under light benzocaine anaesthetic and the eggs were counted. On 329 occasions, gravid females spawned before we could strip and count the eggs, but the spawning events were recorded. Thus, we used the total number of clutches produced throughout the reproductive season as a measure of female fecundity. This fecundity measure was not correlated with individual mean known clutch size (*r*^2^ = 0.008, *F*_1,288_ = 2.225, *P* = 0.137) but strongly correlated with estimated total number of eggs produced per female during the season (calculated as mean known clutch size × number of clutches; *r*^2^ = 0.785, *F*_1,288_ = 1050.7, *P* < 0.001). By the end of August all females in the growth tanks had stopped egg production and most males had become dull, and so we stopped photographing males and monitoring females.

### Quantitative genetic analyses

We estimated additive genetic variances and covariances in male sexual signal (i.e., the seasonal maximum of relative red size), female fecundity (i.e., the total number of clutches) and juvenile survival rate by using pedigree-based restricted maximum likelihood univariate and multivariate animal models implemented in ASReml (version 3). The estimation of the additive genetic (co)variances was based on parental identities. The significance of (co)variance terms was assessed by using model comparison based on likelihood ratio tests.

Male sexual signal and female fecundity were calculated for both individuals and full-sib family replicates (i.e., growth tank means). We first used family replicate means to balance (co)variance component structures of the male and female traits and the family replicate-specific juvenile survival rate. Therefore, in each univariate model a single trait (*t*) of a family replicate, growth tank *gt*, is specified as:

$$ {t}_{gt}=\mu + hatchdate+ exp+{a}_{gt}+{\varepsilon}_{gt}, $$

where *μ* was the overall mean, and hatching date (*hatchdate*) and experimental treatment (*exp*, normal or warm winter schemes) were included as fixed effects. The additive genetic effect (*a*_*gt*_) and the residual error (*ɛ*_*gt*_) were included as random effects. We also fitted a multivariate animal model to test genetic correlations among the three traits. The same fixed and random effects as the univariate model were included in the multivariate model.

We additionally analysed male sexual signal and female fecundity at the individual level in univariate and bivariate animal models, in which any covariance among individuals due to shared environment (growth tank effect) is additionally determined. For example, a single trait (*t*) of an individual *i* is specified as:

$$ {t}_i=\mu + hatchdate+ exp+{a}_i+g{t}_i+{\varepsilon}_i, $$

where the common environment effect (growth tank effect, *gt*_*i*_) was included as an additional random effect.

## Results

In the univariate model analyses based on the family replicate traits, male sexual signal (i.e., seasonal peak relative red area), female fecundity (i.e., number of clutches) and juvenile survival rate showed significant additive genetic effects (Table 1). Hatching date (a fixed term) was significant only in the analysis of male sexual signal (*P* = 0.004) and temperature manipulation only in female fecundity (*P* = 0.013). The males born earlier in the previous year showed a higher peak colouration. The females reared under warm winter conditions produced less clutches than the control females.

**Table 1:** Quantitative genetics of growth tank-based traits (*n* = 114 tanks)

Growth tank traits			Variances			
	No. individuals	Mean ± SD	*V*_A_ ± SE	*V*_P_ ± SE	*h* ^2^ ± SE	*P*
Female fecundity	300	6.136 ± 2.609	1.658 ± 0.825	6.609 ± 0.986	0.251 ± 0.105	0.002
Male signal	209	8.257 ± 2.871	2.799 ± 1.089	7.501 ± 1.207	0.373 ± 0.103	< 0.001
Juvenile survival to maturation	989	0.736 ± 0.205	0.012 ± 0.005	0.042 ± 0.006	0.283 ± 0.104	< 0.001
Covariances	*Cov*_A_ ± SE		*r*_G_ ± SE		*P*	
Female fecundity–juvenile survival	0.119 ± 0.052		0.937 ± 0.188		0.003	
Male signal–juvenile survival	−0.120 ± 0.057		−0.717 ± 0.237		0.015	
Female fecundity–male signal	−0.475 ± 0.673		−0.226 ± 0.321		0.474	

Additive genetic and phenotypic variances (*V*_A_ and *V*_P_) and heritability (*h* ^2^) of female fecundity (number of spawning events), male sexual signal (seasonal maximum of relative red area) and juvenile survival (proportion of individuals that survived to sexual maturation) were calculated by univariate animal model analyses. Additive genetic covariances and correations (*Cov*_A_ and *r*_G_) among the three traits were calculated by a multivariate animal model. The significance of each additive genetic variance or covariance is presented

**Fig. 1:**
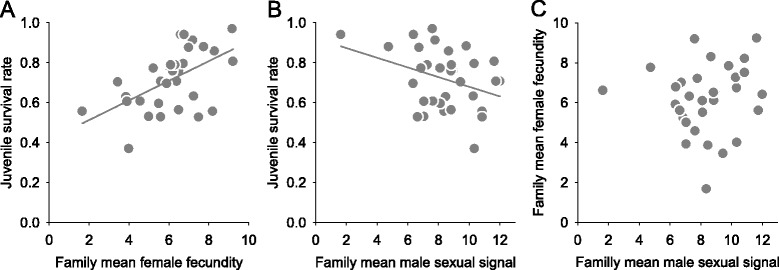
Correlations between full-sib family traits (*n* = 32 families): (**a**) between family mean female fecundity and juvenile survival rate (the proportion of individuals surviving to maturity), (**b**) between family mean male sexual signal (seasonal maximum sexual signal) and juvenile survival rate and (**c**) between family mean male sexual signal and family mean female fecundity. Simple *linear regression lines* are shown for the relationships with a significant genetic correlation (A: *r* ^2^ = 0.304; B: *r* ^2^ = 0.122)

The multivariate model analysis based on the family replicate traits, including trait-specific fixed effects (i.e., hatching date for male sexual signal and temperature manipulation for female fecundity), showed significant genetic correlations between juvenile survival to sexual maturation and reproductive traits (Table 1). Juvenile survival was positively genetically correlated with female fecundity but negatively genetically correlated with male sexual signal. However, there was no significant genetic correlation between female fecundity and male sexual signal (Table 1). The correlations based on full-sib family mean values are presented in Fig. 1 only for the purpose of illustrating the genetic correlation patterns between the three traits.

The analyses based on individual traits confirmed significant heritability in male sexual signal and female fecundity; growth tank effects (*gt*^2^) were not significant in either of the traits (female fecundity: *h*^2^ = 0.196 ± 0.107, *P* = 0.006; *gt*^2^ = 0.052 ± 0.060, *P* = 0.348; male sexual signal: *h*^2^ = 0.499 ± 0.162, *P* < 0.001; *gt*^2^ = 0). The individual-based multivariate analysis fitted to female fecundity and male sexual signal also showed a non-significant intersexual genetic correlation (*r*_G_ = −0.108 ± 0.349, *P* = 0.754).

## Discussion

Our quantitative genetic results show a difference in the way that male sexual attractiveness and female fecundity genetically correlate with juvenile viability, all of which are heritable traits closely related with fitness. There was a strong positive genetic correlation between female fecundity and juvenile viability, suggesting a common genetic basis for variation in these fitness components. In contrast, red sexual signal of male sticklebacks showed a negative genetic correlation with juvenile viability. This result is consistent with a previous finding in another species [23], suggesting that antagonistic pleiotropy between sexual traits and juvenile viability may broadly explain the maintenance of genetic variation under sexual selection. In addition, there was no significant correlation between attractiveness of brothers and fecundity of sisters, suggesting no intra-locus sexual conflict.

The high and significant heritability of carotenoid-based ornament colouration of male sticklebacks supports a previous finding [35] and suggests that choosy females should gain an indirect benefit by producing attractive sons [43, 44]. However, our results show a viability cost of bearing the sexy genes. Similarly, a previous study also showed that sticklebacks sired by redder males were more resistant to parasite infection but grew less quickly than those sired by dull males [34]. The negative effects of mating with a colourful male on offspring viability may contribute to maintaining the heritable variation, necessary for the “good genes” process, despite strong directional sexual selection. Nevertheless, traditional “good genes” predictions are still valid if total expected fitness returns from mating with an attractive male exceed the returns from an unattractive male [45]. Traditional Fisherian models predict negative effects of the expression of costly sexual traits on survival and generally assume that only sexually matured adults bear the costs of attractiveness [6]. Our results suggest that the attractiveness genes may express not only during sexual maturation and reproduction but also during earlier life and their expression has antagonistic effects during the early stage [23, 46].

In sticklebacks, carotenoids are stored in various tissues during development and later mobilized and deposited in the integument during maturation [47]. Perhaps genotypes that store more carotenoids for later use in nuptial colouration pay increased health costs in terms of reduced antioxidant defence and immune function even when not expressing red colouration during the development and growth. The negative genetic correlation between juvenile survival and male sexual signal can also be due to linkage disequilibrium if, for example, deleterious mutations are linked with red colouration in the non-recombining region of Y chromosome [23]. In the three-spined sticklebacks, a nascent Y chromosome has reduced recombination across a region with substantial deletions [48]; selection tends to retain and upregulate some genes in males [49, 50]. However, evidence from diverse animal taxa indicates that the loci underlying sexually selected traits often do not locate in sex chromosome [51].

There was no genetic correlation between male sexual signal and female fecundity. Therefore, the genetic bases of traits underlying male and female reproductive effort probably have little in common. Intersexual genetic correlation is usually large and positive for most homologous traits, but it is often smaller or even negative for fitness components [52–54]. Evidence of intersexual genetic correlation between male secondary sexual signal and female fitness is scarce. However, a study of a field cricket demonstrated that male calling effort was positively genetically correlated with female fecundity, suggesting no intra-locus sexual conflict over reproductive fitness [55]. Similarly, our results suggest that intra-locus sexual conflict will unlikely constrain the evolution of male coloration in this stickleback population.

## Conclusions

The genetic conflict between a male’s sexual signal and other components of fitness is puzzling because there is much evidence of the honesty of carotenoid-based skin or plumage colouration as an indicator of the breeder quality and female preferences for this signal across taxa (e.g., [34, 56–58]). Male sexual signal and female preference of the three-spined stickleback are sexually selected through both increased direct benefits, such as territory quality and paternal care for eggs, and indirect benefits by inheritance of attractiveness and breeder quality [32]. However, the strength of sexual selection may be weaker than previously thought due to the hidden genetic conflict between the secondary sexual trait and viability of juveniles before sexual maturation. The genes involving carotenoid-based animal coloration remain largely unidentified, although some candidate genes have been proposed [57]. The identification and mapping of these genes in male sticklebacks, including those expressed during early development, will improve our understanding of the molecular mechanisms underlying the genetic conflict reported here [58].

### Availability of supporting data

The data set supporting the results of this article are included as an additional file (Additional file 1).

## Additional file

Additional file 1:
**Individual-based traits and growth tank-based traits.** (XLS 53 kb)
